# Comparison of prognostic, clinical, and renal histopathological characteristics of overlapping idiopathic membranous nephropathy and IgA nephropathy versus idiopathic membranous nephropathy

**DOI:** 10.1038/s41598-017-11838-1

**Published:** 2017-09-13

**Authors:** Xinxin Chen, Yu Chen, Keqing Shi, Yinqiu Lv, Huan Tong, Guangju Zhao, Chaosheng Chen, Bo Chen, Duo Li, Zhongqiu Lu

**Affiliations:** 1 Department of Nephrology, The First Affiliated Hospital of Wenzhou Medical University, Wenzhou, Zhejiang Province China; 2 Department of Nephrology, Zhejiang Chinese Medical University Affiliated Wenzhou Hospital of Traditional Chinese Medicine, Wenzhou, Zhejiang Province China; 3 Department of Infectious Disease, The First Affiliated Hospital of Wenzhou Medical University, Wenzhou, Zhejiang Province China; 4 Emergency Department, The First Affiliated Hospital of Wenzhou Medical University, Wenzhou, Zhejiang Province China

## Abstract

Overlapping idiopathic membranous nephropathy (IMN) and immunoglobulin A nephropathy (IgAN) is rare. This study aims to investigate the unique prognostic, clinical, and renal histopathological characteristics of IMN+IgAN. This retrospective observational study included 73 consecutive cases of IMN+IgAN and 425 cases of IMN treated between September 2006 and November 2015. Prognostic and baseline clinical and histopathological data were compared between the two patient groups. Poor prognostic events included a permanent 50% reduction in eGFR, end-stage renal disease, and all-cause mortality. Renal histopathology demonstrated that the patients with IMN+IgAN presented with significantly increased mesangial cell proliferation and matrix expansion, increased inflammatory cell infiltration, and higher proportions of arteriole hyalinosis and lesions than the patients with IMN (all *P* < 0.05). Kaplan–Meier analysis showed that the patients with IMN+IgAN had significantly higher cumulative incidence rates of partial or complete remission (PR or CR, *P* = 0.0085). Multivariate Cox model analysis revealed that old age at biopsy and high baseline serum creatinine and uric acid levels were significantly associated with poor prognosis (all *P* < 0.05), and increased IgA expression correlated significantly with PR or CR (*P* < 0.05). The present study found that overlapping IMN and IgAN presents with unique renal histopathology and appears not to cause a poorer prognosis than IMN.

## Introduction

Membranous nephropathy (MN), an autoimmune glomerular disease with a characteristic renal histopathology of immunoglobulin deposition along the extracapillary side of the glomerular basement membrane, is one of the most common causes for nephrotic syndrome in adults^1–6^. Approximately 75% of patients with MN have idiopathic or primary membranous nephropathy (IMN), and the remaining 25% of cases are associated with various secondary causes^3, 6^. The incidence of MN peaks in the fourth and fifth decades of life and appears higher in men than in women (men: women = 2-3:1)^3–5^. The exact etiology of MN remains unclear, and the disease course varies substantially. Approximately one-third of patients with MN experience spontaneous remission of proteinuria; another one-third develop persistent proteinuria; and the remaining one-third progress to end-stage renal disease (ESRD)^7–9^.

Immunoglobulin A nephropathy (IgAN), also known as Berger’s disease, is the most common primary glomerulonephritis, which is characterized by dominant or codominant IgA mesangial deposition^10, 11^. Although both IMN and IgAN are common primary glomerular diseases, coexisting MN and IgAN in adults is very rare^12–14^. Because of the low incidence, reports on overlapping MN and IgAN are limited to sparse case reports focusing on histopathological features^12–15^. In this study, we retrospectively reviewed 73 cases of overlapping IMN and IgAN and compared the renal histopathology of IMN only versus overlapping IMN and IgAN. In addition, we further examined the follow-up clinical data and compared the prognosis and outcomes of overlapping IMN and IgAN versus IMN only.

## Materials and Methods

### Ethics statement

The study was carried out in accordance with the Declaration of Helsinki and was approved by the Institutional Review Board of The First Affiliated Hospital of Wenzhou Medical University (Approval No: 2016151). All the study participants signed the informed consent form.

### Patient selection

**Figure 1:**
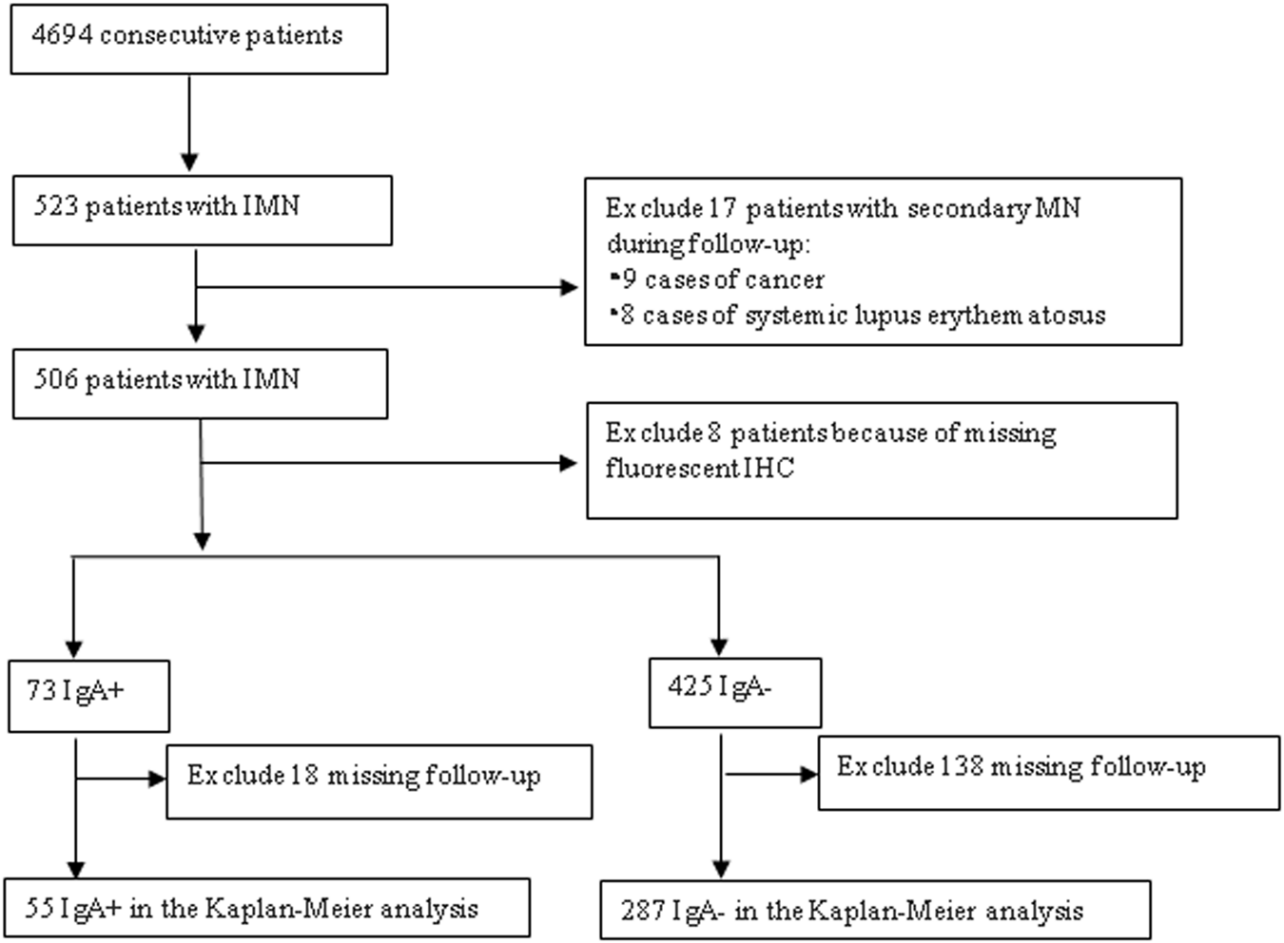
Patient selection flow chart.

A total of 523 patients with IMN were identified from 4694 consecutive patients who underwent renal biopsies and were treated in the Department of Nephrology of The First Affiliated Hospital of Wenzhou Medical University between September 11, 2006 and November 25, 2015. The diagnosis of IMN followed the 2012 KDIGO Clinical Practice Guideline for Glomerulonephritis^16^ and was determined by a careful examination of kidney biopsies using light, immunofluorescence, and electron microscopy and an exclusion of secondary causes for MN. The pathology of the patients’ renal biopsies was characterized by (1) thickened glomerular capillary walls revealed by hematoxylin and eosin (H&E), periodic acid-Schiff (PAS), periodic acid silver methenamine (PASM), and Masson trichrome (Masson) staining; (2) positive IgG and C3 immunofluorescence staining along the capillary walls; and (3) subepithelial deposits and fusion in podocyte structure revealed by electron microscopy^16^. At the initial diagnosis, 523 patients with MN did not present with secondary causes for MN, such as malignancy, systemic lupus erythematosus, hepatitis B or C, diabetic glomerulosclerosis, adverse responses to medications, or renal damage from toxic agents. The authors had access to information that could identify individual participants during or after data collection. However, during follow-up examinations, 17 of the 523 patients were found to have a secondary cause for MN, including 9 cases of cancer and 8 cases of systemic lupus erythematosus. Thus, 506 patients with IMN were analyzed. The flow chart of patient selection is displayed in Fig. 1. Information on patients’ demographics (age and sex), body mass index (BMI, kg/m^2^), course of disease, and comorbidities such as diabetes, hypertension, infections, embolism, edema, nephrotic syndrome, and microscopic hematuria were collected.

### Hematoxylin and eosin (H&E), periodic acid-Schiff (PAS), periodic acid silver methenamine (PASM), and Masson trichrome (Masson) staining

Formalin-fixed paraffin-embedded biopsy tissue specimens were sectioned into 3-μm-thick sections. The tissue sections were stained with H&E, PAS, PASM, and Masson according to the standard protocols. Based on these stains, the extent of mesangial cell proliferation and matrix expansion was graded as 0, 1, 2, and 3, representing negative, mild, moderate, or strong, respectively. In addition, the extent of inflammatory cell infiltration, interstitial fibrosis, tubular atrophy, and renal interstitial lesions were evaluated semi-quantitatively according to a scale 0 to 3, representing very small (scale 0: 0–4%), small (scale 1: 5–24%), medium (scale 2: 25–49%), and large affected area (scale 3: ≥50%). Two pathologists scored the stained sections, and the average score was used. Other histopathological characteristics, including segmental sclerosis, crescent, arteriole hyperplasia, arteriole hyalinosis, and arteriole lesions, were also evaluated.

### Fluorescence immunohistochemical staining for immunoglobulins, complement components, and matrix proteins

The expression of IgG, IgA, IgM, C3, C4, C1q, and fibrinogen was determined by fluorescence immunohistochemical (IHC) staining. Frozen tissue sections (5 μm) were incubated with the following primary antibodies: anti-human IgG (1:50), IgA (1:50), IgM (1:50), C3 (1:50), C4 (1:50), C1q (1:90), and fibrinogen (1:90) at 4 °C overnight. All the antibodies were purchased from Gene Tech Company (Shanghai, China). The tissue sections were washed with PBS buffer and then incubated with FITC-labeled secondary antibodies (1:50, Gene Tech Company, Shanghai, China) at room temperature for 45 minutes. The stained slides were observed under an Olympus BX41 fluorescence microscope (Olympus, Center Valley, PA, USA). Images (200x magnification) were captured using the digital camera attached to the microscope. The intensity of the staining signals was scored as 0, 1, 2, 3, and 4, representing very weak, weak, moderate, strong, and very strong, respectively. IgA positive staining was defined as an intensity score ≥2. The staining intensity was scored by two pathologists, and the average score was used.

### Laboratory test

Urine protein quantification was determined using a 24-hour collection. Nephrotic syndrome was defined as proteinuria ≥3.5 g/d and hypoalbuminemia <30 g/L. Subnephrotic status was defined as >0.5 g proteinuria and <3.5 g per day. Proteinuria was graded as stage 1, 2, 3, and 4 when proteinuria was <3.5 g/d, 3.5–8 g/d, 8–15 g/d, and 15 g/d, respectively. Proteinuria and microscopic hematuria were evaluated by urinary sediment examination. The severity of proteinuria and microscopic hematuria was graded 0–4 with 0 representing the mildest and 4 representing the most severe stage. The estimated glomerular filtration rate (eGFR) was calculated according to the abbreviated Modification of Diet in Renal Disease (MDRD) study formula and serum creatinine levels^17^.

### Electron microscopy

Epon-embedded biopsy tissue specimens were cut into ultrathin sections. The sections were fixed in 2.5% glutaraldehyde and then in 1% osmium tetroxide. The tissue sections were stained with uranyl acetate and lead citrate and examined under a Hitachi H7500 electron microscope (Tokyo, Japan). The ultrastructural stage of MN was determined according to the Ehrenreich and Churg four-stage classification.

### Outcome parameters

The following outcome and prognostic parameters were recorded and used for statistical analysis: development of end-stage renal disease (ESRD), all-cause mortality, a permanent 50% reduction in eGFR compared with the baseline value, complete remission (CR), and partial remission (PR). CR was defined as urinary protein excretion <0.3 g/d (uPCR <300 mg/g or <30 mg/mmol) accompanied by a normal serum albumin concentration and a normal serum creatinine. PR was defined as urinary protein excretion <3.5 g/d (uPCR <3500 mg/g or <350 mg/mmol), a ≥50% reduction from peak values, an improvement or normalization of the serum albumin concentration, and stable serum creatinine^16, 18^. ESRD was defined as a permanent drop in eGFR to <15 mL/min/1.73 m^2^ requiring dialysis or kidney transplantation.

### Statistical analysis

Data are presented as the mean ± standard deviation (SD). Student’s *t*-test and the chi-square test were used for two-group comparisons of continuous variables and categorical variables, respectively. A poor event-free renal survival curve was prepared using the Kaplan-Meier method. Cumulative hazard curves were plotted for CR and PR. Poor event-free renal survival and incidence rates of CR or PR in different groups were compared using the log-rank test. Univariate and multivariate Cox regression analyses were performed to analyze the correlation between the clinical parameters and outcomes. For all variables, the proportional hazard assumption was tested and found to be fulfilled. The statistical analysis software Medcalc version 15.6 was used for the survival analysis, and SPSS version 23 was used for Cox regressions and all other statistical analyses. The *P* value was 2-sided, and *P* < 0.05 was considered statistically significant.

## Results

A total of 506 patients with idiopathic MN identified from the consecutive renal biopsy cases from September 11, 2006 to November 25, 2015 at The First Affiliate Hospital of Wenzhou Medical University were included in this retrospective study. Most patients were treated with RAS receptor inhibitors: angiotensin-converting enzyme inhibitors (ACEis) or angiotensin receptor blockers (ARBs), glucocorticoids, cyclophosphamide, calcineurin inhibitors (CNIs) including tacrolimus and cyclosporine and other immunosuppressors. The usual doses of drugs were as follow: (1) RAS receptor inhibitors: double dosage given orally daily; (2) glucocorticoids plus cyclophosphamide: full oral dosage (1 mg/kg/d) of glucocorticoids per day and i.v. cyclophosphamide 1 g/month until reaching an accumulated dose of 6–8 g for 6 months; (3) CNI therapy (tacrolimus or cyclosporine): tacrolimus 0.05–0.075 mg/kg/d given orally in two divided doses 12 hours apart, without prednisone, for 6–12 months or cyclosporine 3.5–5.0 mg/kg/d given orally in two equally divided doses 12 hours apart, with prednisone 0.15 mg/kg/d, for 6 months.

Because of the use of glucocorticoids and immunosuppressors, 24 patients experienced infections during the follow-up period: 20 infections were respiratory tract infections, particularly pulmonary infections; 1 was a lower limb skin infection; 1 patient had both a respiratory and lower limb skin infection; 1 patient had tuberculotic hydrothorax; 1 patient had Hepatitis E virus infection; 1 patient was infected with neurosyphilis; and 2 patients died because of serious infections. Five patients had embolic events during the follow-up period including 4 cerebral infarctions and 1 mesenteric arterial embolism. One patient suffered a subarachnoid hemorrhage and 1 patient had an upper gastrointestinal hemorrhage secondary to a duodenal ulcer. Four patients had repetitive renal biopsies. One patient’s twin brother also had nephrotic syndrome. Two patients had renal biopsies done during pregnancy.

### Patients with IgA+ nephropathy presented with higher mesangial cell proliferation and matrix expansion but lower segmental sclerosis than patients with IgA− nephropathy

Of the 506 patients with IMN, 8 were excluded because of missing fluorescent IHC data. There were 73 cases of IgA+ and 425 cases of IgA− in the remaining 498 patients. Overall, 18 patients in the IgA+ group and 138 patients in the IgA− group did not have a follow-up examination after 6 months. Thus, 55 cases of IgA+ and 287 cases of IgA− were included in the outcome and prognosis analyses. The patient selection flow chart is displayed in Fig. 1.

Demographics including gender distribution, age at disease onset, and BMI were similar in the IgA+ and IgA− groups (Table 1). The proportions of patients with comorbidities, such as edema, hypertension, diabetes, infection, embolism, nephrotic syndrome, and/or microscopic hematuria, were also similar in the two groups. Serum IgA levels were significantly higher in the IgA+ group than in the IgA− group (2.44 ± 0.95 g/L vs. 2.15 ± 0.89 g/L, *P* = 0.011, Table 1). All the other serum and urine test results were comparable in the two groups (Table 1).

**Table 1:** Baseline clinical characteristics.

	IgA+, N = 73	IgA−, N = 425	*P* value
**Demographics**			
Men, n (%)	38 (52.1)	229 (53.9)	0.772
Age at disease onset, (years)	49.7 ± 12.4	49.9 ± 15.6	0.938
Body mass index, (kg/m^2^)	24.1 ± 2.8	24.7 ± 12.0	0.663
**Comorbidities, n (%)**			
Edema	58 (79.5)	348 (81.9)	0.621
Hypertension	33 (45.2)	190 (44.7)	0.937
Diabetes	8 (11.0)	44 (10.4)	0.876
Infection	10 (13.7)	74 (17.4)	0.434
Embolism	0 (0.0)	19 (4.5)	0.131
Nephrotic syndrome	37 (50.7)	247 (58.1)	0.236
Microscopic hematuria	29 (39.7)	175 (41.2)	0.816
**Serum albumin, Immunoglobulin, Complement**			
D-dimer (mg/L)	1.43 ± 1.33	1.81 ± 2.61	0.264
Fibrinogen (g/L)	4.94 ± 3.95	4.82 ± 2.25	0.702
ESR (mm/h)	28.08 ± 16.88	31.64 ± 19.93	0.290
Serum albumin (g/L)	25.30 ± 7.16	24.33 ± 6.41	0.281
Immune globulin G (g/L)	7.18 ± 3.20	6.95 ± 2.73	0.569
Immune globulin A (g/L)	**2.44 ± 0.95**	**2.15 ± 0.89**	**0.011**
Immune globulin M (g/L)	1.28 ± 0.57	1.40 ± 0.77	0.236
Complement 3 (g/L)	1.00 ± 0.21	1.01 ± 0.21	0.670
Complement 4 (g/L)	0.23 ± 0.07	0.24 ± 0.08	0.391
**Renal function indicators**			
Serum creatinine (μmol/L)	67.60 ± 23.01	67.60 ± 23.29	1.000
Blood urea nitrogen (mmol/L)	5.34 ± 2.41	5.15 ± 2.21	0.515
Serum uric acid (μmol/L)	358.74 ± 79.81	377.46 ± 95.32	0.114
eGFR (mL/min/1.73 m^2^)	109.69 ± 28.61	112.06 ± 32.28	0.496
**Serum mineral levels**			
Calcium (mmol/L)	2.00 ± 0.14	1.99 ± 0.13	0.552
Phosphorus (mmol/L)	1.18 ± 0.18	1.19 ± 0.20	0.653
**Urine test**			
Urine specific gravity	1.017 ± 0.00	1.015 ± 0.01	0.097
Proteinuria severity	2.40 ± 0.83	2.52 ± 0.80	0.230
Urine protein quantification (g/24 h)	5.14 ± 4.05	5.72 ± 4.09	0.263
Urine protein quantification stage	1.84 ± 0.87	1.92 ± 0.84	0.458

ESR: Erythrocyte sedimentation rate; eGFR: estimated glomerular filtration rate by abbreviated MDRD equation. Data are presented as the mean ± standard deviation or number of cases (% of total cases). Student’s *t*-test or chi-square test was used to compare the values of the two groups.

Examination of the renal biopsies revealed that patients with IgA+ had significantly higher pathological stages compared with patients with IgA− (1.88 ± 0.73 vs. 1.65 ± 0.57, *P* = 0.003, Table 2). In particular, the IgA+ group had significantly increased mesangial cell proliferation, matrix expansion, and inflammatory cell infiltration than the IgA− group (All *P* < 0.05, Table 2). In contrast, the proportion of patients with segmental sclerosis was significantly lower in the IgA+ group than in the IgA− group (4.7% vs. 12.9%, *P* = 0.009, Table 2). In addition, significantly higher proportions of patients with IgA+ than patients with IgA− exhibited arteriole hyalinosis (41.1% vs. 25.4%, *P* = 0.006) and lesions (58.9% vs. 37.4%, *P* = 0.001, Table 2). Patients with IgA+ also appeared more likely to have arteriole hyperplasia than patients with IgA− (32.9% vs. 22.4%, *P* = 0.051, Table 2). Fluorescence IHC showed positive IgG for both groups and positive IgA for the IgA+ group. The staining intensity for IgM, C3, C4, C1q, and fibrinogen were weak, although the IgA+ group showed significantly stronger staining for IgM than the IgA− group (0.62 ± 0.90 vs. 0.32 ± 0.65, *P* = 0.008, Table 2).

**Table 2:** Baseline histopathological examination and fluorescence immunohistochemical staining of renal biopsies.

	IgA+, N = 73	IgA−, N = 425	*P* value
**Histopathological characteristics (severity score)**			
Pathological stage	1.88 ± 0.73	1.65 ± 0.57	**0.003**
Mesangial cell proliferation	0.82 ± 0.82	0.36 ± 0.65	**<0.001**
Mesangial matrix expansion	0.96 ± 0.90	0.59 ± 0.86	**0.001**
Segmental sclerosis n, (%)	18 (4.7%)	55 (12.9%)	**0.009**
Crescent n, (%)	4 (5.5%)	17 (4.0%)	0.790
Inflammatory cell infiltration	0.86 ± 0.54	0.72 ± 0.57	**0.043**
Interstitial fibrosis	0.71 ± 0.61	0.60 ± 0.61	0.153
Tubular atrophy	0.78 ± 0.58	0.72 ± 0.57	0.404
Renal interstitial lesions stage	0.78 ± 0.58	0.68 ± 0.59	0.183
Arteriole hyperplasia n, (%)	24 (32.9%)	95 (22.4%)	0.051
Arteriole hyalinosis n, (%)	30 (41.1%)	108 (25.4%)	**0.006**
Arteriole lesions n, (%)	43 (58.9%)	159 (37.4%)	**0.001**
**Fluorescence immunohistochemical staining (staining intensity score)**			
IgG	2.84 ± 0.41	2.79 ± 0.54	0.520
IgA	2.26 ± 0.44	0.04 ± 0.18	**<0.001**
IgM	0.62 ± 0.90	0.32 ± 0.65	**0.008**
C3	1.77 ± 0.91	1.59 ± 0.87	0.119
C4	0.24 ± 0.56	0.08 ± 0.33	0.059
C1q	0.37 ± 0.67	0.33 ± 0.61	0.622
Fibrinogen	0.40 ± 0.85	0.26 ± 0.60	0.199

Data are presented as the mean ± standard deviation of severity score or staining intensity or the number of positive cases (% of total cases). Student’s *t*-test or the chi-square test was used to compare the values of the two groups.

**Figure 2:**
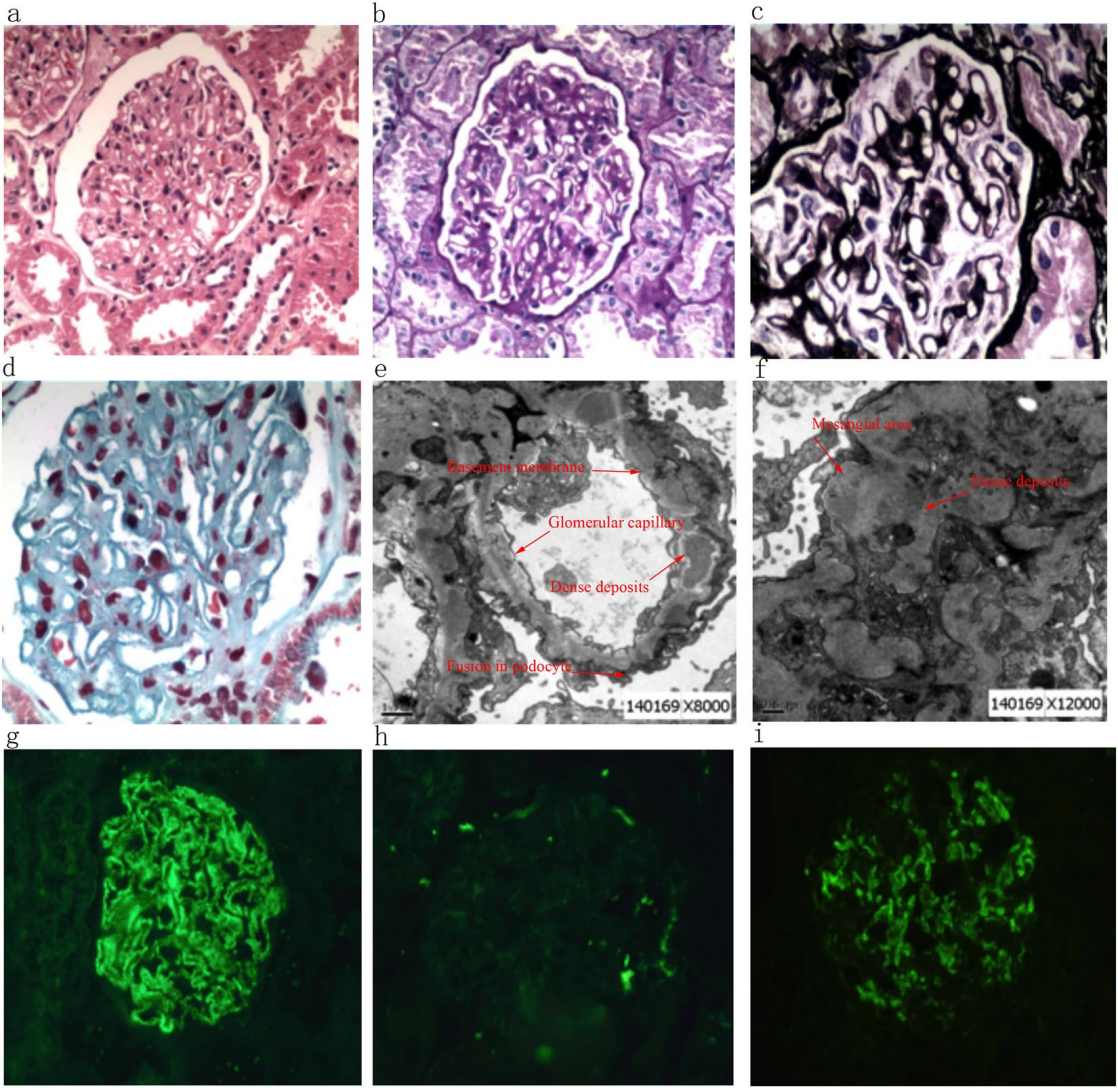
Representative images of the renal biopsy histopathology of patients with IgA+ at pathological stage II-III. (**a**) H&E staining (200x). (**b**) PAS staining (200x). (**c**) PASM staining (200x). (**d**) Masson staining (200x). (**e** and **f**). Electron microscopy. (**g**) Immunofluorescence staining for IgG (200x). (**h**) Immunofluorescence staining for C3 (200x). (**i**) Immunofluorescence staining for IgA (200x).

**Figure 3:**
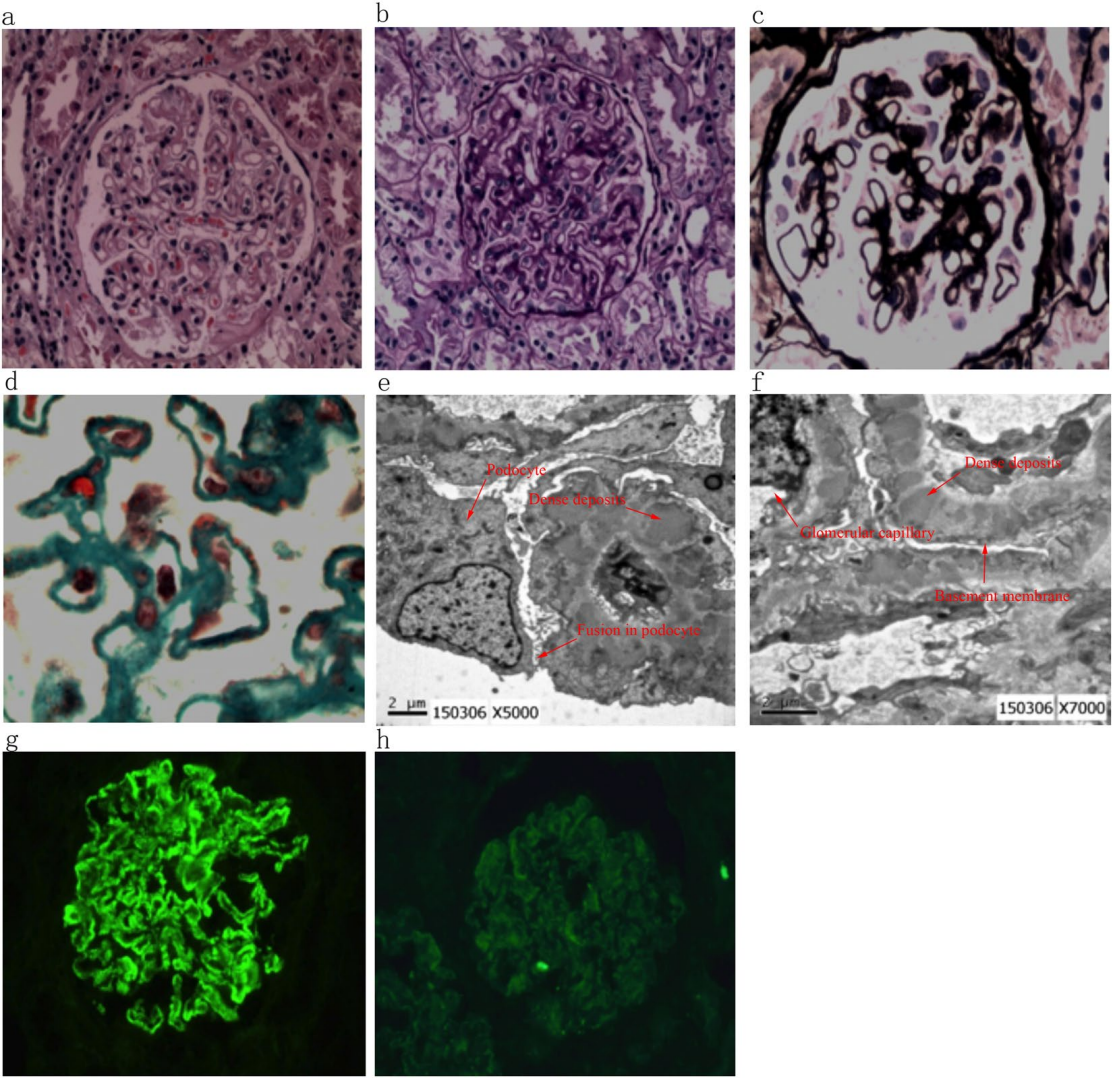
Representative images of the renal biopsy histopathology of patients with IgA− at pathological stage II-III. (**a**) H&E staining (200x). (**b**) PAS staining (200x). (**c**) PASM staining (200x). (**d)** Masson staining (400x). (**e** and **f**). Electron microscopy. (**g**) Immunofluorescence staining for IgG (200x). (**h**) Immunofluorescence staining for C3 (200x).

Representative images of H&E, PAS, PASM, Masson, and fluorescence staining and representative images of electron microscopy for the IgA+ and IgA− groups are presented in Figs 2 and 3. Patients with IgA+ showed stiff capillary loops on H&E (Fig. 2a **)** and PAS staining **(**Fig. 2b), spikes in the peripheral capillary wall on the PASM staining (Fig. 2c), and protein deposition (red colored area) on the peripheral capillary wall on the Masson staining (Fig. 2d). Electron microscopy showed global irregularities; capillary wall thickening; dense electron deposits in the sub-epithelium, intramembranous space, and mesangial areas; and epithelial foot process effacement (Figs. 2e and f). Patients at stages II-III had higher levels of mesangial cell proliferation and matrix expansion (Fig. 2), stronger staining for IgG (Fig. 2g), and more obvious epithelial foot process effacement (Fig. 2e and f) than patients at a lower pathological stage (stage I-II). Strong IgA staining was detected in patients in the IgA+ group at all pathological stages (Fig. 2). The staining signals of IgG appeared predominantly on the peripheral capillary walls, whereas the staining signals of IgA were mainly found in the mesangial area. The renal histopathology of patients with IgA− showed an absence of mesangial cell proliferation and matrix expansion (Fig. 3).

### Patients with IgA+ showed significantly higher cumulative incidence rates of PR or CR than patients with IgA−

The follow-up duration ranged from 6 to 111 months. The IgA− group had a significantly longer follow-up duration than the IgA+ group (18.57 ± 16.32 months vs. 12.79 ± 12.03 months, *P* = 0.001, Table 3). At the latest follow-up examination, BUN, SCr, serum UA and albumin levels, proteinuria severity, and hematuria severity were similar in the two groups (Table 3).

**Table 3:** Clinical data from the latest follow-up examination.

	IgA+, N = 73	IgA−, N = 425	*P* value
Follow-up duration (months)	12.79 ± 12.03	18.57 ± 16.32	**0.001**
BUN (mmol/L)	6.00 ± 3.40	6.24 ± 3.95	0.660
SCr (μmol/L)	74.28 ± 34.33	87.22 ± 104.33	0.075
Serum UA (μmol/L)	342.62 ± 99.98	370.45 ± 101.26	0.052
Albumin (g/L)	34.35 ± 9.27	34.20 ± 8.58	0.900
Proteinuria severity	1.76 ± 1.40	1.90 ± 1.39	0.460
Hematuria severity	0.52 ± 0.75	0.64 ± 0.97	0.248

BUN: Blood urea nitrogen; SCr: serum creatinine; UA: uric acid. Data are presented as the mean ± standard deviation. Student’s *t*-test was used to compare the values of the two groups.

**Figure 4:**
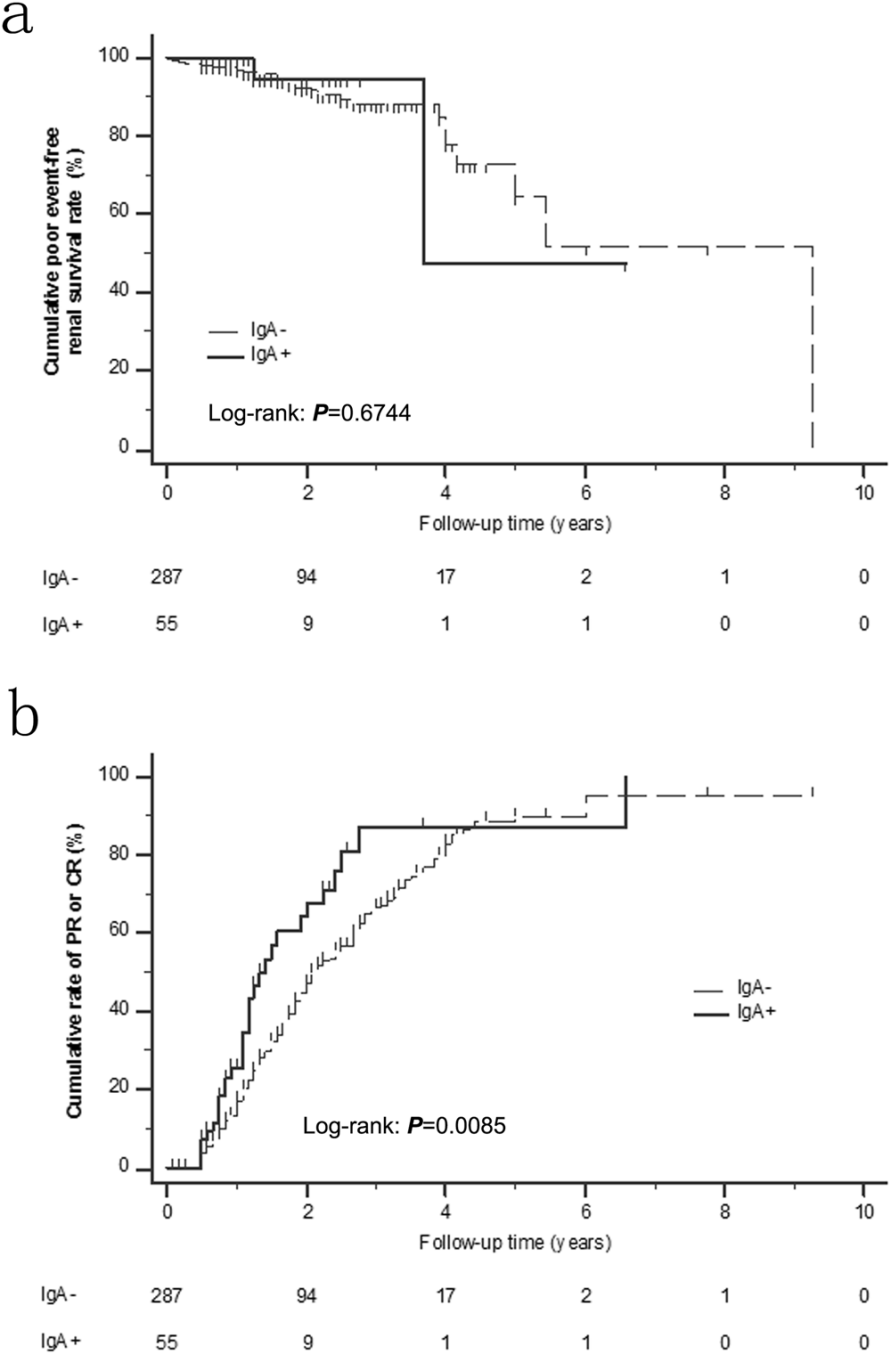
Kaplan-Meier analysis. (**a**) Cumulative poor event-free renal survival rate in all patients (including with treatment and without treatment) between the IgA+ and IgA− groups. (**b**) Cumulative incidence rates of PR or CR in all patients (including patients with and without treatment) between the IgA+ and IgA− groups.

To compare the prognostic disease characteristics of the two patient groups, we performed Kaplan-Meier (K-M) analyses to investigate the cumulative poor event-free renal survival and cumulative incidence rates of PR or CR. Patients with longer than 6 months of follow-up duration, including 55 cases of IgA+ and 287 cases of IgA−, were included for the K-M analyses. A poor event was defined as an occurrence of a 50% reduction in eGFR, ESRD, or all-cause mortality. The one-year and 4-year cumulative poor event-free renal survival rates were 100.0% and 47.4%, respectively, in patients with IgA+, and they were 96.9% and 78%, respectively, in patients with IgA− (Fig. 4a). Although the 4-year survival rates were lower in the IgA+ group (47.4%) than in the IgA− group (78%), the differences were not statistically significant (odds ratio: 1.355; 95% CI: 0.3804–4.8234; *P* = 0.6744). Notably, the K-M analysis also revealed that the one, two, and three-year cumulative incidence rates of PR or CR were 25.6%, 67.7%, and 87.2%, respectively, in patients with IgA+, whereas the rates were 17.3%, 47.0%, and 66.4%, respectively in patients with IgA− (Fig. 4b). The patients with IgA+ had significantly higher cumulative incidence rates of PR or CR than patients with IgA− (odds ratio: 1.653; 95% CI: 1.0331–2.6441; *P* = 0.0085).

**Figure 5:**
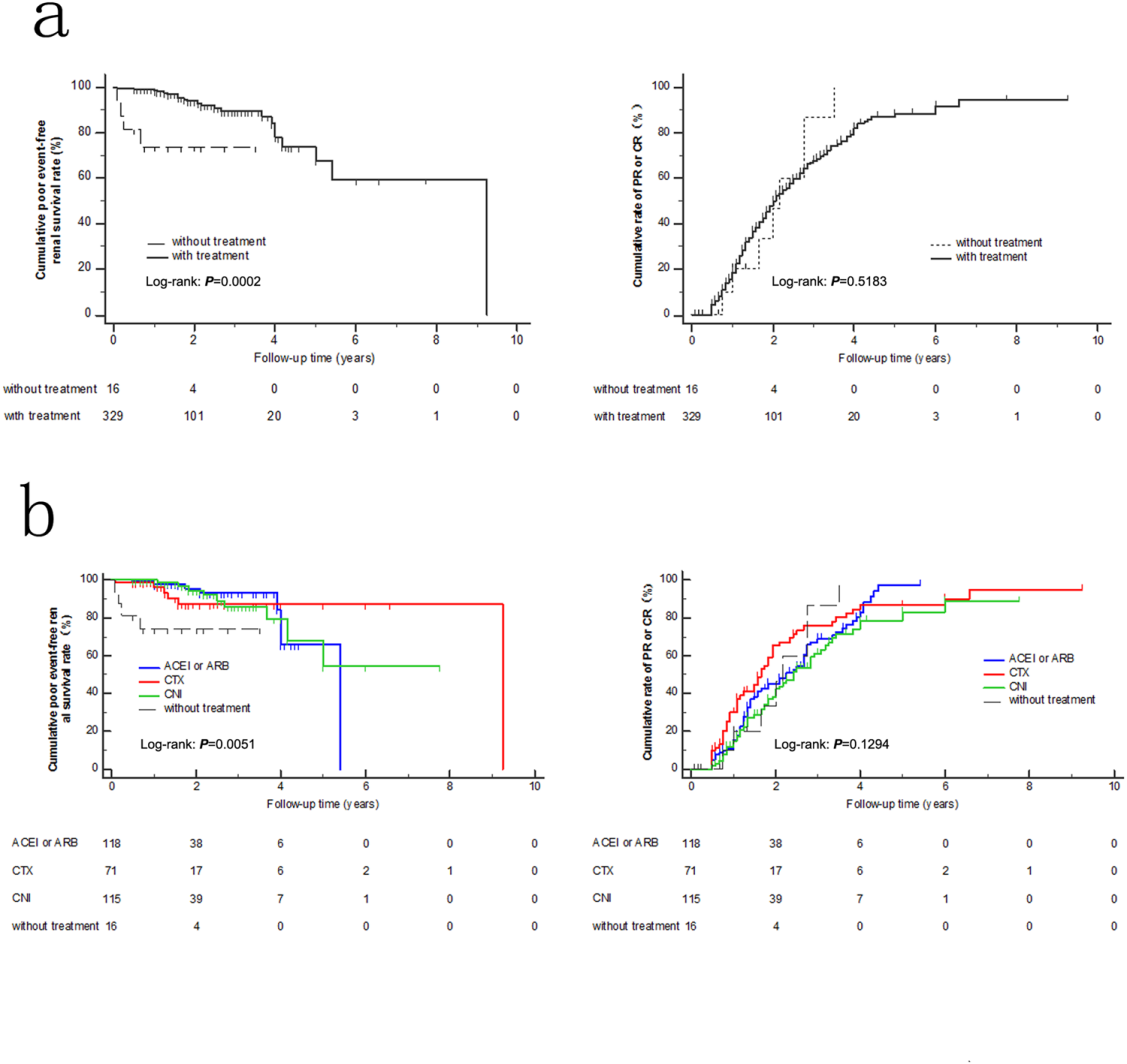
Kaplan-Meier analysis. (**a**) Cumulative poor event-free renal survival rate and cumulative incidence rates of PR or CR in all patients with and without treatment. (**b)** Cumulative poor event-free renal survival rate and cumulative incidence rates of PR or CR in all patients receiving different treatments.

We next considered treatments; we performed K-M analysis to compare the prognosis with and without treatment. The with treatment group had a higher poor event-free renal survival rate than the without treatment group (*P* = 0.0002) (Fig. 5a). The different treatment groups had different poor prognoses (*P* = 0.0051), but good prognoses among the different treatment groups were negative (Fig. 5b).

Next, we doubted that different treatments could influence IgA+ and IgA− groups. We classified similar cohorts into four groups as a whole, including (1) without treatment: general treatment without RAS inhibitors or glucocorticoids or immunosuppressors; (2) RAS receptor inhibitors; (3) glucocorticoids plus cyclophosphamide (GC + CTX); and (4) CNI therapy (tacrolimus or cyclosporine). There were no treatment differences between the IgA+ and IgA− groups (*P* = 0.517) as a whole: 3 (5.5%) versus 13 (4.5%) between the IgA+ and IgA− groups (*P* = 0.758) without treatment; 22 (40.0%) versus 96 (33.1%) between the IgA+ and IgA− groups with RAS receptor inhibitor treatment (*P* = 0.323); 13 (23.6%) versus 58 (20.0%) between the IgA+ and IgA− groups (*P* = 0.541) receiving treatment with GC + CTXt; and 14 (25.5%) versus 101 (34.8%) between the IgA+ and IgA− groups (*P* = 0.176) under CNI treatment (Table 4). Therefore, there was no statistical difference between treatments, including whole and partial; therefore, we can evaluate IgA’s deposition independent of the different treatments.

**Table 4:** Different treatments among patients with at least 6 months of follow-up between IgA+ and IgA− groups.

Different treatments	IgA+, N = 55	IgA−, N = 290	*P* value	
Without treatment	3 (5.5%)	13 (4.5%)	0.758	0.517
RAS receptor inhibitors	22 (40.0%)	96 (33.1%)	0.323	
GC + CTX	13 (23.6%)	58 (20.0%)	0.541	
CNIs	14 (25.5%)	101 (34.8%)	0.176	

(1) Without treatment: general treatment without RAS inhibitors or glucocorticoids or immunosuppressors; 2) RAS receptor inhibitors: double dosage given orally per day, ACEIs or ARBs; 3) Glucocorticoids plus cyclophosphamide (GC + CTX): oral full dosage (1 mg/kg/d) of glucocorticoids per day and i.v. cyclophosphamide 1 g/month until accumulated dose of 6–8 g for 6 months; 4) CNI therapy (tacrolimus or cyclosporine): tacrolimus 0.05–0.075 mg/kg/d given orally in two divided doses 12 hours apart, without prednisone, for 6–12 months or cyclosporine 3.5–5.0 mg/kg/d given orally in two equally divided doses 12 hours apart, with prednisone 0.15 mg/kg/d, for 6 months. Data are presented as the number of positive cases (% of total cases). Chi-square test was used to compare the values of the two groups.

### Association of baseline clinical parameters and disease prognosis

Of the 506 patients, 342 had a follow-up duration >6 months. We then focused on these 342 patients and carried out multivariate Cox regression analyses to identify factors that could be significantly associated with a poor prognosis or the incidence of PR or CR. Poor prognosis was defined as an occurrence of a 50% reduction in eGFR, ESRD, or all-cause mortality. Multivariate Cox analyses revealed that old age at biopsy, high baseline SCr and serum UA were significantly associated with a poor prognosis (Table 5). The multivariate Cox analysis also found that increased interstitial fibrosis and IgA expression, and reduced hypertension, BUN, and proteinuria were significantly associated with an incidence of PR or CR (Table 6). Additionally, the univariate Cox analysis revealed that the different treatments were not associated with either a good or a poor prognosis (Tables S1 and S2).

**Table 5:** The association between clinical parameters and poor prognosis. Factors predicting prognosis in univariate and multivariate Cox regression models.

Factor	Univariate Cox Model		Multivariate Cox Model	
	Hazard ratio (95% CI)	*P* value	Hazard ratio (95% CI)	*P* value
Age at biopsy	1.067 (1.032–1.103)	**<**0.001	1.114 (1.024–1.212)	0.012
Infection	2.970 (1.321–6.678)	0.008		
Nephrotic syndrome	4.689 (1.609–13.665)	0.005		
ALB	0.941 (0.887–0.998)	0.044		
SCr	1.028 (1.018–1.039)	**<**0.001	1.061 (1.032–1.090)	**<**0.001
UA	1.004 (1.000–1.007)	0.035	1.011 (1.005–1.016)	**<**0.001
Proteinuria	2.034 (1.130–3.662)	0.018		
Urine protein quantification	1.088 (1.014–1.167)	0.019		

Poor prognosis was defined as an occurrence of a permanent 50% reduction in eGFR, ESRD, or all-cause mortality. A multivariate Cox model was used for the correlation analysis. *P* < 0.05 was considered statistically significant. ALB: albumin. SCr: serum creatinine. UA: uric acid. CI: confidence interval.

**Table 6:** The association between clinical parameters and a good prognosis. Factors predicting prognosis in univariate and multivariate Cox regression models.

Factors	Univariate Cox Model		Multivariate Cox Model	
	Hazard ratio (95% CI)	*P* value	Hazard ratio (95% CI)	*P* value
Hypertension	0.569 (0.421–0.770)	0.001	0.591 (0.425–0.821)	0.002
BUN	0.883 (0.812–0.960)	0.004	0.893 (0.809–0.985)	0.023
Proteinuria	0.750 (0.635–0.886)	0.001	0.816 (0.680–0.979)	0.029
Interstitial fibrosis	1.551 (1.212–1.985)	**<**0.001	2.509 (1.296–4.857)	0.006
Renal interstitial lesions stage	1.349 (1.040–1.752)	0.024		
IgA expression	1.300 (1.091–1.549)	0.003	1.234 (1.025–1.485)	0.026

Good prognosis was defined as an occurrence of PR or CR. Multivariate Cox model was used for the correlation analysis. *P* < 0.05 was considered statistically significant. BUN: blood urea nitrogen; CI: confidence interval.

## Discussion

Overlapping MN and IgAN is considered to be a rare medical condition. The majority of previous studies on this rare condition were reports of a single case or a case series and focused on a description of the renal pathology^12–15, 19^. Notably, the current study included the largest patient cohort of overlapping IMN and IgAN (73 cases) and IMN alone (425 cases), which compared the prognostic, clinical, and histopathological characteristics of the two patient groups. Similar to the findings in the current study, the previous reports have also shown that IgG deposits are mainly in the capillary walls, while IgA deposits are predominantly in the mesangial area in the renal pathology of patients with overlapping MN and IgAN^12–15, 19^.

In the current study, we found similar baseline clinical features in the overlapping IMN and IgAN groups and the IMN only group. Although the current study showed a statistically significantly higher pathological stage in the IgA+ group than in the IgA− group, the increment was only approximately 14% (1.88 vs. 1.65) and therefore may represent a minimal clinical significance.

To the best of our knowledge, the current study is the first comprehensive comparison of the renal pathological features of overlapping IMN and IgAN versus IMN only and to define the unique renal histopathology associated with overlapping IMN and IgAN. Compared with the IMN only group, the renal histopathology of the overlapping IMN and IgAN groups not only exhibited significantly increased mesangial cell proliferation and matrix expansion, which are characteristic of the renal histopathology of IgAN but also showed increased inflammatory cell infiltration and higher proportions of arteriole hyalinosis and lesions. However, the proportion of segmental sclerosis was significantly lower in the overlapping IMN and IgAN group compared with the IMN alone group.

The lower chance of segmental sclerosis in the patients with overlapping IMN and IgAN may explain the high incidence of PR or CR in these patients. In the current study, we performed K-M analysis to discover that the one, two, and three-year cumulative incidence rates of PR or CR were significantly higher in the patients with overlapping IMN and IgAN than in the patients with IMN only. Consistently, the multivariate Cox model analysis also revealed that increased IgA expression was significantly associated with the incidence of PR or CR. PR and CR have been recommended as surrogate endpoints to evaluate the outcomes of patients with IMN^20^. Although the cumulative poor event-free renal survival rate was lower in the patients with overlapping IMN and IgAN than in the patients with IMN alone, the difference was not statistically significant. A previous case series study showed a similar renal prognosis in overlapping MN and IgAN versus MN only^12^. Stokes *et al*. also indicated that the combination of MN and IgAN may not lead to a poorer clinical outcome compared with MN alone^12^. Similarly, Nishida *et al*. reported a pediatric case of coexisting MN and IgAN and suggested that overlapping MN and IgAN might not lead to deleterious outcomes^21^. The physiological and cellular mechanisms underlying the overlapping IMN and IgAN-associated increase in disease remission remains unclear.

To avoid the effects of different treatments between overlapping IMN and IgAN and IMN only, we took treatment into account. We found that there was a better prognosis with drug treatment than without treatment, and the prognosis rank differed between different drug treatments.

However, despite the different drug treatments, poor prognosis displayed no significant statistical difference between overlapping IMN and IgAN and IMN only. We speculate that the reason there is no difference is because the different treatments in our research were chosen according to the clinical symptoms, such as persistent nephrotic syndrome, rather than pathological features such as the presence or absence of IgA deposition, which is also recommended by the KDIGO clinical practice guidelines for glomerulonephritis of IMN^16^. The proportions of patients with comorbidities, such as edema, hypertension, diabetes, infection, embolism, nephrotic syndrome, and/or microscopic hematuria, were similar between the IgA+ and IgA− groups. In other words, the clinical symptoms were comparable between the IgA+ and IgA− groups, and therefore the treatments were also comparable between the IgA+ and IgA− groups. Therefore, we can evaluate IgA’s deposition independent of the different treatments.

The etiology of overlapping IMN and IgAN is unlikely to be due to chance alone. IMN and IgAN could develop simultaneously, or either IgAN or IMN could be the primary event and subsequently stimulate the other nephropathy^19^. Miyazaki *et al*. reported a case of the development of IgAN 14 years after the diagnosis of MN^22^. Genetic factors may contribute to the development of overlapping IgAN and MN. Familial overlapping of IgAN and MN has been reported previously^19, 23, 24^.

The current study also found that increased age at biopsy and high baseline SCr and serum UA levels significantly increased the risk of a 50% reduction in eGFR, ESRD, or all-cause mortality. Similarly, recent studies have also demonstrated that persistent nephrotic range proteinuria and elevated creatinine levels are associated with poor outcomes in patients with IMN^25, 26^. The correlation between high interstitial fibrosis and the incidence of PR or CR may be explained by the interstitial fibrosis-mediated nonspecific reduction in urine secretion. We speculate that increased interstitial fibrosis may result in the blockage of capillary loops and the reduction in urinary protein excretion, therefore leading to a false sense of a better prognosis.

The main limitation of the current study is that the follow-up duration of patients varied greatly, which results in big variations in the renal survival analysis. We are currently continuing to follow up with the patients and will further investigate the prognostic features of overlapping IMN and IgAN. The M-type phospholipase A2 receptor (PLA2R) has mostly been studied in IMN since 2009. However, because our patient selection occurred between September 11, 2006 and November 25, 2015 in this research and the commercial detection of PLA2R in the clinic was not mature during this time in our district, PLA2R was not a consideration in our study.

## Conclusions

This retrospective observational study found that, in addition to presenting the IgAN-characteristic renal histopathology, such as significantly increased mesangial cell proliferation and matrix expansion, patients with overlapping IMN and IgAN also exhibited unique features, such as increased inflammatory cell infiltration, higher proportions of arteriole hyalinosis and lesions, but lower proportions of segmental sclerosis compared with patients with IMN only. In addition, overlapping IMN and IgAN appears to correlate with an increased incidence of disease remission and thus may not lead to a poorer prognosis compared with IMN only.

## Electronic supplementary material

Supplement infromation
