# Supplementary material for: Comparison of prognostic, clinical, and renal histopathological characteristics of overlapping idiopathic membranous nephropathy and IgA nephropathy versus idiopathic membranous nephropathy
Source: Sci Rep. 2017 Sep 13;7:11468. doi: 10.1038/s41598-017-11838-1 (PMC5597578; doi:10.1038/s41598-017-11838-1)
Supplement: Supplementary file 1 — Supplement infromation [file 41598_2017_11838_MOESM1_ESM.pdf]

Comparison of prognostic, clinical, and renal histopathological characteristics of overlapping idiopathic membranous nephropathy and IgA nephropathy versus idiopathic membranous nephropathy

Xinxin Chen[1], Yu Chen[2], Keqing Shi[3], Yinqiu Lv[1], Huan Tong[4], Guangju Zhao[4], Chaosheng Chen[1], Bo Chen[1], Duo Li[1], Zhongqiu Lu[4]*

Supporting information:

Table S1. The association between different treatments and poor prognosis. Factors predicting prognosis in univariate Cox regression models.

Factor	Univariate Cox Model	
	Hazard ratio (95%CI)	*P* value
Different treatment	0.698 (0.472-1.033)	0.072

Poor prognosis was defined as an occurrence of a permanent 50% reduction in eGFR, ESRD, or all-cause mortality. $P < 0.05$ was considered statistically significant. CI: confidence interval.

Table S2. The association between different treatments and good prognosis. Factors predicting prognosis in univariate Cox regression models.

Factors	Univariate Cox Model	
	Hazard ratio (95%CI)	*P* value
Different treatment	0.927 (0.811-1.061)	0.270

Good prognosis was defined as an occurrence of PR or CR. $P < 0.05$ was considered statistically significant. CI: confidence interval.
